# Agreement and workflow efficiency of AI-based coronary artery calcification quantification in lung cancer screening: Comparison with semi-automated and visual assessment

**DOI:** 10.1016/j.ejro.2026.100799

**Published:** 2026-07-18

**Authors:** Katharina Ochs, Falko Ensle, Jasmin Happe, Lisa Jungblut, Thomas Frauenfelder, Jonas Kroschke

**Affiliations:** Diagnostic and Interventional Radiology, University Hospital Zurich, University of Zurich, Zurich, Switzerland

**Keywords:** Coronary Artery Calcification, Lung Cancer Screening, Low-Dose Computed Tomography, Artificial Intelligence, Agatston Score, Opportunistic Screening, Workflow Efficiency

## Abstract

**Objectives:**

To evaluate agreement and workflow implications of fully automated AI-based coronary artery calcification (CAC) quantification on non-ECG-gated low-dose CT in lung cancer screening, compared with semi-automated (SA) and visual assessment.

**Materials and methods:**

In this retrospective single-center study, 323 participants (55.7% male; median age 61 years; 52–79 years) undergoing low-dose CT for lung cancer screening were included. CAC was quantified using SA and AI-based Agatston scoring. Two readers performed visual grading. Agreement between SA and AI was assessed using intraclass correlation coefficient (ICC), Spearman correlation, and Bland-Altman analysis. Categorical agreement (CAD-RADS 2.0 plaque burden) and CAC detection were evaluated using weighted Cohen’s κ and diagnostic metrics. CAC processing times for were compared.

**Results:**

AI-based and semi-automated Agatston scores showed excellent agreement (ICC 0.96) and strong correlation (Spearman r = 0.97), with a small bias (21.5) and moderate limits of agreement. AI achieved high diagnostic performance for excluding CAC (sensitivity 0.97, 95%-CI, 0.92–0.99; specificity 0.91, 95%-CI, 0.86–0.94). Categorical agreement between AI and SA was almost perfect (κ 0.92) and higher than agreement between SA and visual assessment (κ 0.84 and 0.68). SA scoring required substantially longer processing time (102.0 ± 95.7 s) compared with visual assessment (15.5 ± 6.0 s and 24.2 ± 7.4 s; *p* < 0.001).

**Conclusion:**

AI-based CAC quantification on non-ECG-gated low-dose CT demonstrates excellent agreement compared to semi-automated scoring, with higher categorical agreement than visual assessment and no requirement for manual scoring. AI-based approaches may facilitate standardized and scalable CAC reporting in lung cancer screening without additional reading time.

## Highlights

• AI accurately quantifies CAC on non-gated low-dose screening CT.
• AI shows higher agreement with SA scoring than visual assessment.
• Automated CAC scoring may improve efficiency in lung cancer screening.

## 1 Introduction

Lung cancer screening using low-dose computed tomography (CT) has been implemented in several countries following large-scale trials demonstrating a significant reduction in lung cancer-related mortality among high-risk populations [1], [2]. Risk factors for lung cancer, particularly cigarette smoking, overlap with those for cardiovascular disease. As a result, coronary artery calcification (CAC) represents a frequent and clinically relevant comorbidity that can be detected on screening chest CT examinations [3], [4]. Current guidelines from leading societies involved in lung cancer screening recommend routine reporting of CAC, given its well-established association with adverse cardiovascular outcomes and increased overall mortality [5], [6].

While ECG-gated CT remains the reference standard for CAC quantification, multiple studies have demonstrated the feasibility of assessing CAC on non–ECG-gated chest CT, albeit with certain technical limitations [7], [8], [9], [10]. Consequently, guideline recommendations include both (semi-)automated quantification using Agatston scoring and/or visual assessment approaches for CAC evaluation on routine chest CT [11], [12]. However, semi-automated quantification remains time-consuming and requires dedicated user interaction, limiting its routine use in high-throughput screening settings. In contrast, visual assessment is rapid and easily integrated into clinical workflows but is subject to interobserver variability and reduced reproducibility [13], [14]. Recent advances in artificial intelligence (AI) have enabled fully automated CAC quantification directly from non-ECG-gated chest CT, offering the potential to combine high accuracy with improved efficiency and standardization [15], [16], [17]. In the present study, we evaluated a commercially available, deep-learning solution designed for fully automated CAC quantification on non-ECG-gated chest CT. The software was selected because it was integrated into the institutional PACS workflow and had previously undergone external technical validation [15].

Despite these promising developments, the clinical performance of AI-based CAC quantification in comparison with established semi-automated and visual approaches, as well as its potential impact on workflow efficiency, remains incompletely defined in the context of lung cancer screening. The aim of this study was to validate an AI-based CAC quantification method on non-ECG gated LDCT against semi-automated Agatston scoring as reference standard in a lung cancer screening cohort and to evaluate potential efficiency gains and agreement relative to visual scoring.

## 2 Materials and methods

### 2.1 Patient cohort

This retrospective cross-sectional study was conducted at a single tertiary academic center within an ongoing lung cancer screening pilot study. Ethics approval was obtained from the local institutional review board (KEK-ZH-Nr. 2019–01676), and the study was conducted in accordance with the principles of the Declaration of Helsinki. The requirement for written informed consent was waived due to the retrospective study design.

**Fig. 1:**
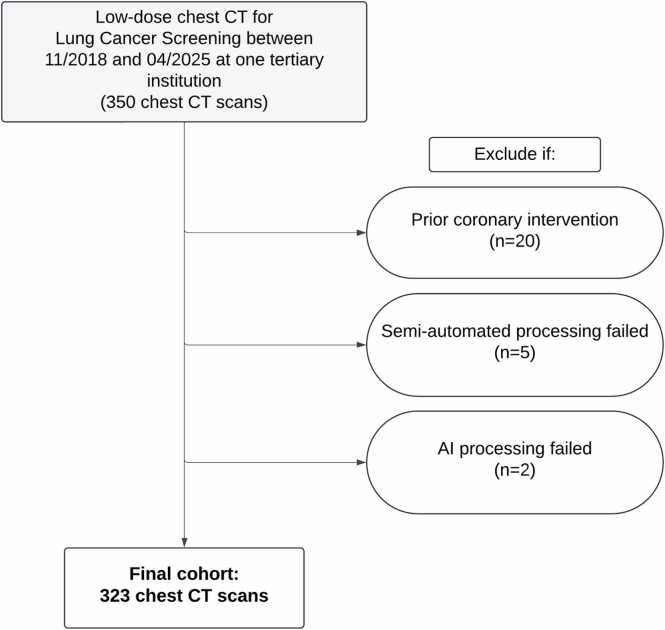
Patient flowchart.

The analysis included previously acquired non-ECG-gated unenhanced low-dose chest CT examinations performed between November 2018 and April 2025. No additional imaging was performed, and no additional radiation exposure was incurred for the purpose of this study. Consecutive participants undergoing lung cancer screening during the study period were eligible for inclusion (n = 350). Exclusion criteria comprised prior coronary intervention as well as failed semi-automated or AI-based processing, leading to a final cohort of 323 scans (Fig. 1).

### 2.2 CT examination criteria

Examinations were conducted on three different scanners: Siemens NAEOTOM Alpha (171 scans), Siemens SOMATOM Edge Plus (130 scans) and Siemens SOMATOM Force (22 scans) (Siemens Healthineers, Forchheim, Germany). Standardized imaging protocols were used across all scanners with a tube voltage of 100 or 120 kVp and tin filtration. Images were reconstructed in the axial plane using a medium-smooth soft tissue kernel (Br36 for NAEOTOM Alpha and SOMATOM Force and Br38 for SOMATOM Edge Plus). A standard slice thickness of 2 mm with an increment of 1 mm was selected.

### 2.3 Semi-automated and AI-based coronary calcification assessment

Semi-automated CAC quantification was performed by a resident radiologist (3 years of experience in cardiac imaging) using a commercially available cardiac analysis software (syngo.via, version VB60, Siemens Healthineers, Erlangen, Germany) under the supervision of a board-certified radiologist (8 years of experience). Automated calcium detection and classification to the corresponding coronary arteries was performed by the software. Automated results were then validated by the resident radiologist. The supervising board-certified radiologist initially reviewed a subset of examinations with the resident to ensure standardized application of the scoring procedure. Subsequently, cases with uncertain segmentation, calcium classification, or coronary vessel assignment were reviewed and adjudicated together. The finalized semi-automated scores were used as the within-scan reference method. Semi-automated assessment was performed before AI analysis and was therefore blinded to the AI-derived results.

AI-based CAC quantification was performed using commercially available, FDA-cleared and CE-marked software (ClearRead CT CAC, release 1.2.1.90; Riverain Technologies, Miamisburg, OH, USA) designed for fully automated CAC detection and Agatston score calculation on non–ECG-gated chest CT, including low-dose examinations. According to the technical description, the underlying deep-learning model uses a U-Net-like encoder–decoder architecture with multislice context to segment coronary calcifications and derive Agatston scores and was trained on approximately 2000 heterogeneous chest CT examinations [15], [18].

The analysis for this study was performed automatically in the background, and the results were transferred directly to PACS as a DICOM report containing the Agatston score and representative screenshots. Exact background processing time was not recorded because the results were available in PACS when the examination was opened for reporting and required no user interaction. Two examinations were excluded because no AI output was generated. The precise cause could not be determined retrospectively but was considered most likely related to the local PACS or software-interface integration rather than to an erroneous algorithmic result.

For comparison with visual scoring, SA- and AI-derived CAC scores were stratified into groups of severity in accordance with CAD-RADS 2.0 reporting recommendations [19] (supplementary Table S1).

### 2.4 Visual coronary artery calcification assessment

Visual quantification of coronary artery calcifications was performed by two readers (with 3 and 7 years of experience in cardiac imaging) using an ordinal scoring method as described by Shemesh et al. [7]. Both readers were blinded to the semi-automated and AI-derived results during visual assessment. In this approach, each coronary artery was scored separately, and the total score was calculated as the sum of the per-vessel scores. For ordinal comparison between methods, the four visual categories (absent, mild, moderate, and severe) were aligned with the four Agatston-based plaque-burden categories used for semi-automated and AI-based assessment [19], as previously described [20]. A detailed overview of the rating categories is provided in the Supplementary Material (Table S1).

### 2.5 Time assessment

To evaluate the time efficiency of each CAC assessment approach, processing times were recorded. For SA assessment, total processing time was recorded after the study was loaded and automatically preprocessed and stopped, once manual validation/correction was completed. Accordingly, reading times for visual assessment were recorded separately for each reader, starting once the corresponding imaging study was loaded into PACS. AI analysis was initiated automatically after image reconstruction and performed in the background on the institution’s integrated server infrastructure without reader interaction. The resulting Agatston score, plaque-burden category, and DICOM report were transferred automatically to PACS and were available when the examination was opened for reporting. Exact background processing duration and server specifications were not recorded.

### 2.6 Statistical analysis

Statistical analysis was performed using Python (SciPy library, version 1.16.3 [21]). Unless otherwise specified continuous variables are presented as mean ± standard deviation, median, and range, and categorical variables as counts and percentages. Data normality was assessed using the Shapiro-Wilk test. Depending on distribution, either paired *t*-tests (for normally distributed data) or Wilcoxon signed-rank tests (for non-normally distributed data) were used to compare paired observations. A two-tailed p-value of < 0.05 was considered statistically significant.

CAC detection agreement between SA- and the AI-based approach was evaluated using confusion‑matrix metrics (sensitivity, specificity, positive predictive value - PPV, negative predictive value - NPV and accuracy) and Cohen’s kappa. Association of continuous Agatston scores between SA and AI was assessed using the intraclass correlation coefficient (ICC; two-way mixed-effects, absolute agreement, single measures), Spearman correlation, scatter plots against the line of identity, and Bland-Altman analysis (log-transformed using log(score + 1) due to skewing of Agatston scores).

For categorical plaque‑burden and visual CAC scores, inter‑method and inter‑reader agreement was quantified using weighted Cohen’s kappa and results were interpreted according to Landis and Koch [22].

## 3 Results

Characteristics of the final cohort are summarized in Table 1.

**Table 1:** Summary of patient characteristics.

**Characteristics**	
Total (n)	323
Age, median, (range) [years]	61 (52−79)
Sex, male/female (%)	180 (55.7) / 143 (44.3)

### 3.1 AI-based vs. semi-automated agatston-scoring

**Fig. 2:**
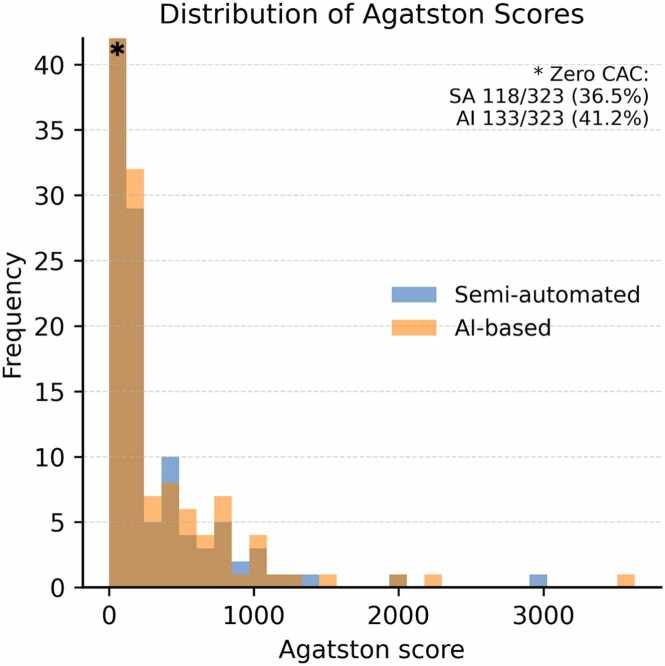
Histograms showing the distribution of Agatston scores for Semi-automated (SA, light blue) and AI-based (AI, yellow) coronary artery calcification scoring. NB: y-axis was truncated for improved readability. Counts of scans with no detected CAC are therefore provided separately (“Zero CAC”).

Agatston scores showed a similar distribution for both semi-automated and AI-based assessments (Fig. 2). Mean Agatston scores still differed significantly, averaging 116.6 ± 289.5 for semi-automated scoring and 138.1 ± 343.9 for AI-based scoring (p < 0.001).

**Fig. 3:**
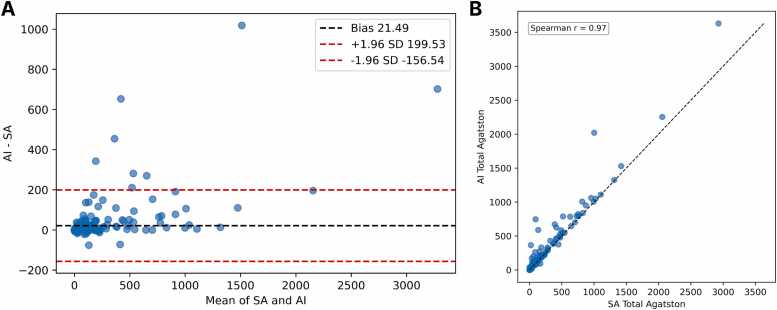
Agreement and correlation between AI-based and semi-automated Agatston scoring. A: Bland-Altman analysis shows a bias of 21.5 with 95% limits of agreement from −156.5 to 199.5, with increasing variability at higher scores. Outliers were primarily attributable to AI-misclassification of non-coronary calcifications (e.g. aortic/valvular calcifications). B: Strong correlation between methods (Spearman r = 0.97), with the dashed line indicating the line of identity.

Bland-Altman analysis showed a mean difference of + 21.49, with 95% limits of agreement ranging from −156.54 to 199.53 (Fig. 3). To account for the skewed distribution and assess agreement on a relative scale, additional BA-analysis was performed after log-transformation, which confirmed no relevant proportional bias but increased variability at low CAC values (bias 0.04, LOA −0.94 and 1.01; supplementary Figure S1). Eight examinations (2.5%) showed differences above the upper Bland–Altman limit of agreement, whereas none showed differences below the lower limit. All therefore represented AI overestimation. Targeted review identified erroneous inclusion of non-coronary calcifications, predominantly involving the aortic valve or root and the mitral annulus, as the principal cause. Five of these examinations remained in plaque-burden category 3 with both methods, while three were reclassified upward by AI, including two from category 1 to category 3 and one from category 2 to category 3.

Strong correlation between SA and AI scores was observed (Spearman r = 0.97, p < 0.001) (Fig. 3) and agreement between AI-based and semi-automated Agatston scoring was excellent (ICC = 0.96; 95% CI, 0.95–0.97).

### 3.2 Performance of AI-based exclusion of coronary calcium

The AI-based approach for the exclusion of CAC demonstrated high diagnostic performance, using semi-automated quantification as the reference standard. For the exclusion of CAC, sensitivity was 0.97 (95% CI, 0.92–0.99) and specificity was 0.91 (95% CI, 0.86–0.94). The positive predictive value was 0.86 (95% CI, 0.79–0.91), the negative predictive value was 0.98 (95% CI, 0.95–0.99), and overall accuracy was 0.93 (95% CI, 0.90–0.95) (Table 2). These findings indicate that the algorithm can identify examinations without CAC with high confidence, supporting a potential role in workflow optimization or cohort preselection in large imaging datasets.

**Table 2:** Performance Metrics for AI-based Exclusion of Coronary Artery Calcification.

**Performance Metrics for CAC Exclusion**		
		**95% CI**
Sensitivity	0.97	0.92–0.99
Specificity	0.91	0.86–0.94
Positive Predictive Value (PPV)	0.86	0.79–0.91
Negative Predictive Value (NPV)	0.98	0.95–0.99
Accuracy	0.93	0.90–0.95

### 3.3 Comparison of AI-based and visual approaches with semi-automated assessment as reference

**Fig. 4:**
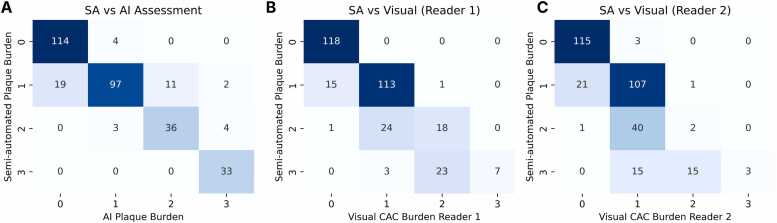
Confusion Matrices comparing AI-based (A) and visual assessments (B, C) against semi-automated assessment (SA). Rows represent semi-automated plaque-burden categories and columns represent AI-based or visual categories. Categories 0–3 correspond to Agatston scores of 0, 1–99, 100–299, and ≥ 300, respectively.

When mapped to CAD-RADS™ 2.0 plaque burden categories (0, 1–99, 100–299, ≥300), AI-based and semi-automated scoring assigned 280 of 323 examinations (86.7%) to the same category. Of the 43 discordant classifications, 41 (95.3%) involved adjacent categories (Fig. 4). Discordance was most frequent at the lower end of the plaque-burden spectrum: 23 of 43 cases (53.5%) occurred between categories 0 and 1, including 19 examinations classified as category 1 by semi-automated scoring but as category 0 by AI and four examinations classified as category 0 by semi-automated scoring but as category 1 by AI. Fourteen discordant cases occurred between categories 1 and 2, four between categories 2 and 3, and two differed by two categories, with semi-automated category 1 classified as AI category 3. AI assigned a lower category than semi-automated scoring in 22 cases and a higher category in 21 cases. All 33 examinations classified as category 3 by semi-automated scoring were also classified as category 3 by AI. Overall agreement between AI-derived and semi-automated CAC categories was almost perfect (weighted κ = 0.92; 95% CI, 0.89–0.95).

Agreement between semi-automated scoring and visual assessment was lower but remained strong, demonstrating almost perfect agreement with reader 1 (weighted κ = 0.84; 95% CI, 0.80–0.88) and substantial agreement with reader 2 (weighted κ = 0.68; 95% CI, 0.62–0.74). Interreader agreement was almost perfect (weighted κ = 0.85; 95% CI, 0.81–0.89).

### 3.4 CAC assessment times

Mean processing time for semi-automated scoring was 102.0 ± 95.7 s per examination. Visual assessment required 15.5 ± 6.0 s for Reader 1 and 24.2 ± 7.4 s for Reader 2.

**Fig. 5:**
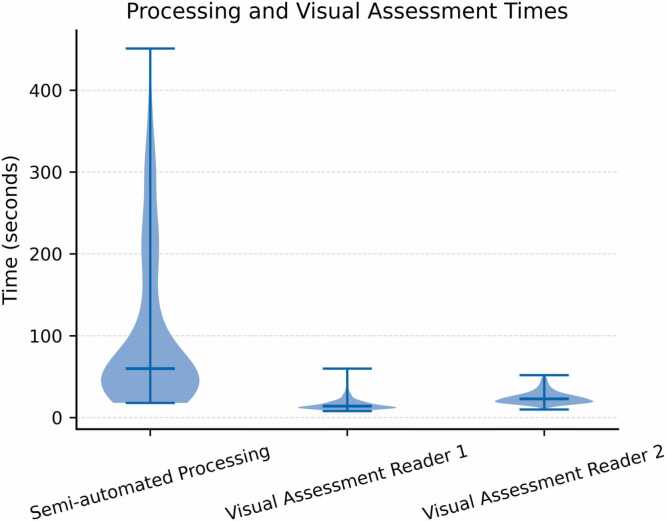
Processing and Assessment Time Comparison. Violin plot illustrating distribution of processing and assessment times for semi-automated scoring and visual CAC grading. Semi-automated scoring required significantly longer processing time compared with visual assessment (p < 0.001).

Processing time differences between semi-automated and visual scoring were statistically significant (all p < 0.001) (Fig. 5). Semi-automated processing demonstrated substantial variability, with occasional prolonged processing times, whereas visual assessment showed a narrower distribution of assessment durations.

AI-based CAC quantification was performed automatically without active reader interaction and therefore did not require manual processing time.

## 4 Discussion

In this study, we evaluated a fully automated AI-based approach for CAC quantification on non-ECG-gated low-dose CT in a lung cancer screening cohort. There are three main findings: first, AI-based CAC quantification demonstrated excellent agreement with semi-automated Agatston scoring for both continuous values and categorical plaque burden, considered the reference standard in this study; second, AI-based classification showed higher concordance with the reference standard than visual assessment; and third, fully automated analysis enables substantial workflow efficiency gains by eliminating the need for manual interaction.

CAC detected on non-gated chest CT is a well-established marker of cardiovascular risk and is associated with both cardiovascular events and all-cause mortality in lung cancer screening populations. In this setting, reliable and efficient CAC assessment is essential to maximize the clinical value of screening examinations. Our results demonstrate that AI-based quantification provides robust and accurate CAC assessment, with excellent agreement with semi-automated Agatston scoring (ICC 0.96), while the strong Spearman correlation (r = 0.97) indicated consistent ranking across the range of CAC burden. However, the raw Bland–Altman limits of agreement demonstrate that exact continuous scores are not interchangeable at the individual-examination level. Given that CAC assessment in lung cancer screening is primarily intended to identify and categorize plaque burden rather than reproduce an identical numerical score, the almost perfect categorical agreement (weighted κ = 0.92) is particularly relevant to the intended clinical application.

Importantly, AI-based classification into CAD-RADS plaque burden categories showed almost perfect agreement with semi-automated scoring (κ = 0.92), indicating that clinically relevant categorization can be reliably achieved using automated analysis. The distribution of discordant classifications provides important context beyond the overall agreement statistic. Most disagreements occurred near category boundaries, particularly between the absence of CAC and low plaque burden, and may therefore affect whether CAC is reported as absent or present. In contrast, large numerical differences were uncommon and exclusively reflected AI overestimation, most often caused by erroneous inclusion of aortic valvular or root calcifications and mitral annular calcifications. Most of these outliers did not alter plaque-burden categorization because both methods yielded scores of ≥ 300; however, three resulted in upward reclassification. Overall, six examinations assigned to lower plaque-burden categories by semi-automated scoring were classified as category 3 by AI, whereas none of the examinations assigned to category 3 by semi-automated scoring were underestimated. These findings identify occasional overestimation of adjacent non-coronary calcifications as a specific but infrequent failure mode. Prospective studies incorporating cardiovascular risk factors and downstream management are required to determine the clinical consequences of these discordant classifications.

Agreement between semi-automated and visual assessment was lower, although still substantial. This finding is consistent with prior literature demonstrating that visual CAC scoring, while rapid and easily integrated into routine workflows, is inherently limited by interobserver variability and reduced reproducibility [13], [14]. These limitations may be particularly relevant in longitudinal follow-up and risk stratification, where consistent quantification is essential.

A notable strength of the AI-based approach is its high diagnostic performance for CAC exclusion. The absence of CAC is associated with a very low risk of cardiovascular events, making reliable identification of CAC-negative individuals clinically important. In our study, the AI algorithm achieved high specificity (0.97) and positive predictive value (0.98), indicating that examinations classified as CAC-negative can be excluded with high confidence. This supports the potential use of AI-based CAC exclusion as a triage tool for automated workflow optimization and cohort preselection in large screening populations.

The excellent agreement observed across three scanner models, two tube-voltage settings, and different reconstruction kernels suggests that the algorithm is robust to the acquisition variability encountered in routine lung cancer screening and supports its potential for real-world implementation. Nevertheless, because all examinations originated from a single institution and scanners from one manufacturer, additional multicenter and multivendor studies are required to establish broader generalizability.

Workflow efficiency represents another key advantage of AI-based CAC quantification. Semi-automated scoring required substantial processing time and manual interaction, whereas visual assessment was significantly faster but reader-dependent. In contrast, AI-based analysis is performed automatically in the background, with results available for review without dedicated manual scoring. Although misclassifications were overall infrequent, they indicate that visual verification of AI-derived results may be required. The AI-based approach therefore addresses an important barrier to routine CAC quantification in high-throughput screening settings, although the need for at least limited human oversight should be considered when estimating real-world efficiency gains.

Our findings are consistent with prior studies demonstrating excellent performance of automated CAC quantification, while extending these observations to a lung cancer screening setting. Kim et al. reported near-perfect agreement of deep-learning-based CAC scoring in both ECG-gated and non-gated CT (ICC up to 0.997, κ up to 0.97) [16], and Hamelink et al. similarly showed excellent agreement between AI-based and manual scoring (ICC > 0.99, κ ≈ 0.90–0.95) [23]. Importantly, the recent study by McKinney et al. evaluating the CAC algorithm used in this study, demonstrated similarly strong performance on non-gated CT compared to the gold standard of matched ECG-gated scans [15].

While these previous studies have largely focused on technical validation or comparisons with gated CT reference standards, our study directly compared automated, semi-automated, and visual CAC assessment in a lung cancer screening cohort while also considering workflow requirements. Semi-automated Agatston scoring provides quantitative results but is generally impractical for routine screening because manual verification and correction are time-consuming; in our study, it required a mean of 102 s of active user interaction per examination and therefore served primarily as a quantitative within-examination reference method. Visual assessment is faster and readily incorporated into screening reports but is limited to ordinal categories and depends on reader interpretation. In contrast, the AI approach automatically generates Agatston scores and standardized plaque-burden categories, with results directly available in PACS without manual segmentation, vessel assignment, or a separate validation step, thereby requiring no additional assessment time. This may enable systematic quantitative CAC reporting in high-volume lung cancer screening programs and support more consistent assessment across readers and serial examinations. Although occasional misclassification of non-coronary calcifications occurred, categorical agreement with semi-automated scoring remained high. The clinical role of AI in lung cancer screening is therefore not to replace routinely performed semi-automated scoring, which is rarely feasible in this setting, but to provide quantitative CAC information that would otherwise generally be limited to visual categorization.

Some limitations should be considered. Semi-automated Agatston scoring on non-ECG-gated low-dose CT was used as the within-examination reference method rather than dedicated ECG-gated cardiac CT, which remains the gold standard for absolute Agatston score quantification. Accordingly, our findings demonstrate agreement between assessment methods on the same screening examination but do not establish absolute accuracy relative to ECG-gated CT. Additionally, CAC measurements may be influenced by acquisition and reconstruction parameters, including tube voltage and scanner type. Because AI-based and semi-automated assessments were performed on identical image datasets, these effects were shared by both methods; nevertheless, protocol heterogeneity should be considered when extrapolating the results to other acquisition settings. This was a single-center study evaluating one commercially available AI tool, which limits generalizability. Examinations with failed semi-automated or AI-based processing were excluded; therefore, the reported performance applies to successfully analyzable scans and does not reflect full end-to-end applicability. Misclassification of non-coronary calcifications contributed to several outliers, although categorical plaque-burden classification was largely preserved. Finally, outcome data were unavailable, precluding assessment of the prognostic implications of AI-derived CAC measurements.

Fully automated AI-based CAC quantification on non-ECG-gated low-dose CT demonstrates excellent agreement with semi-automated Agatston scoring and provides accurate plaque burden classification while reducing the need for dedicated manual scoring. By combining high diagnostic performance with potential efficiency gains, AI-based approaches may help enable standardized and scalable CAC reporting in lung cancer screening, thereby enhancing the clinical utility of incidental cardiovascular findings.

## CRediT authorship contribution statement

**Falko Ensle:** Writing – review & editing, Writing – original draft, Methodology, Investigation, Formal analysis, Data curation. **Jonas Kroschke:** Writing – review & editing, Writing – original draft, Visualization, Validation, Project administration, Methodology, Investigation, Formal analysis, Data curation, Conceptualization. **Thomas Frauenfelder:** Writing – review & editing, Visualization, Validation, Supervision, Software, Resources, Project administration, Methodology, Investigation, Conceptualization. **Katharina Ochs:** Writing – original draft, Methodology, Investigation, Formal analysis, Data curation. **Lisa Jungblut:** Writing – review & editing, Validation, Methodology, Formal analysis, Data curation. **Jasmin Happe:** Writing – review & editing, Writing – original draft, Investigation, Formal analysis, Data curation.

## Declaration of Generative AI and AI-assisted technologies in the writing process

During the preparation of this work the authors used ChatGPT solely for the purpose of language editing. After using this tool/service, the authors reviewed and edited the content as needed and take full responsibility for the content of the published article.

## Declaration of Competing Interest

The authors declare that they have no known competing financial interests or personal relationships that could have appeared to influence the work reported in this paper.
