# Supplementary material for: Agreement and workflow efficiency of AI-based coronary artery calcification quantification in lung cancer screening: Comparison with semi-automated and visual assessment
Source: Eur J Radiol Open. 2026 Jul 18;17:100799. doi: 10.1016/j.ejro.2026.100799 (PMC13400849; doi:10.1016/j.ejro.2026.100799)
Supplement: Supplementary file 1 — Supplementary material [file mmc1.docx]

Supplements

**Table S1:** Overview of the Visual Assessment Criteria for Subjective Calcium Scoring^7^ and Comparison with Agatston scores.

| **Agatston Score** | **Visual Assessment** | | |
| --- | --- | --- | --- |
|  | **Likert Score** | Individual Artery (Shemesh-Score) | Overall  (Shemesh-Score) |
| - | 0 | Absent | Absent |
| 1-100 | 1 | Mild: <1/3 of length of coronary artery shows calcifications | Mild (1-3) |
| 101-300 | 2 | Moderate: 1/3-2/3 of length of artery shows calcifications | Moderate (4-7) |
| >300 | 3 | Severe: > 2/3 of length of artery shows calcifications | Severe (8-12) |

**Figure S1:** Log-transformed Bland-Altman analysis shows minimal bias (black line) and good limits of agreement (red lines) between AI-based and semi-automated Agatston scoring, with no relevant proportional bias and increased variability at low CAC values.

**
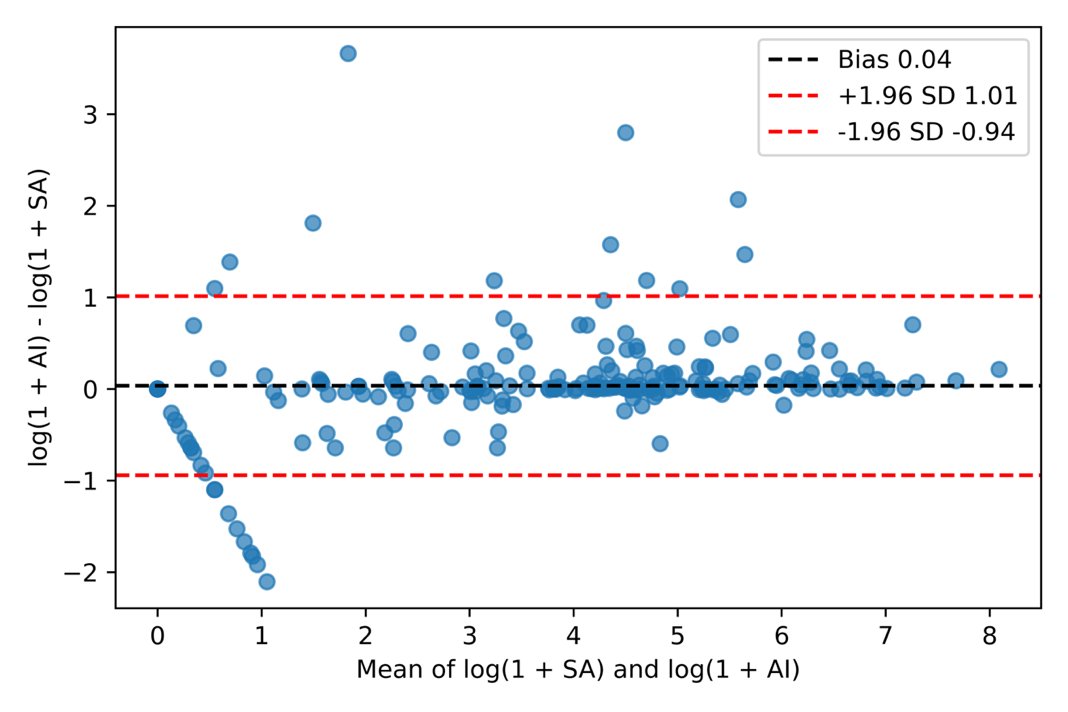
**
